# Supplementary material for: A scalable workflow to characterize the human exposome
Source: Nat Commun. 2021 Sep 22;12:5575. doi: 10.1038/s41467-021-25840-9 (PMC8458492; doi:10.1038/s41467-021-25840-9)
Supplement: Supplementary file 1 — Supplementary Information [file 41467_2021_25840_MOESM1_ESM.pdf]

Supplementary Information:
A scalable workflow to characterize the human exposome

Xin Hu[1], Douglas I. Walker[2], Yongliang Liang[1], M. Ryan Smith[1], Michael L. Orr[1], Brian D. Juran[3], Chunyu Ma[4], Karan Uppal[1], Michael Koval[1], Greg S. Martin[1], David C. Neujahr[1], Carmen J. Marsit[5], Young-Mi Go[1], Kurt Pennell[6], Gary W. Miller[7], Konstantinos N. Lazaridis[3], Dean P. Jones[1]

[1]Division of Pulmonary, Allergy, Critical Care, and Sleep Medicine, Department of Medicine, School of Medicine at Emory University, Atlanta, GA;

[2]Department of Environmental Medicine and Public Health, Icahn School of Medicine at Mount Sinai, New York, NY;

[3]Division of Gastroenterology and Hepatology, Mayo Clinic, Rochester, MN;

[4]Huck Institute of the Life Sciences, Penn State University

[5]Department of Environmental Health, Rollins School of Public Health at Emory University, Atlanta, GA;

[6]School of Engineering, Brown University, Providence, RI;

[7]Department of Environmental Health Sciences, Mailman School of Public Health, Columbia University, New York, NY.

*Corresponding author; dpjones@emory.edu

615 Michael St. 205P Whitehead Building, Emory University, Atlanta GA, 30322

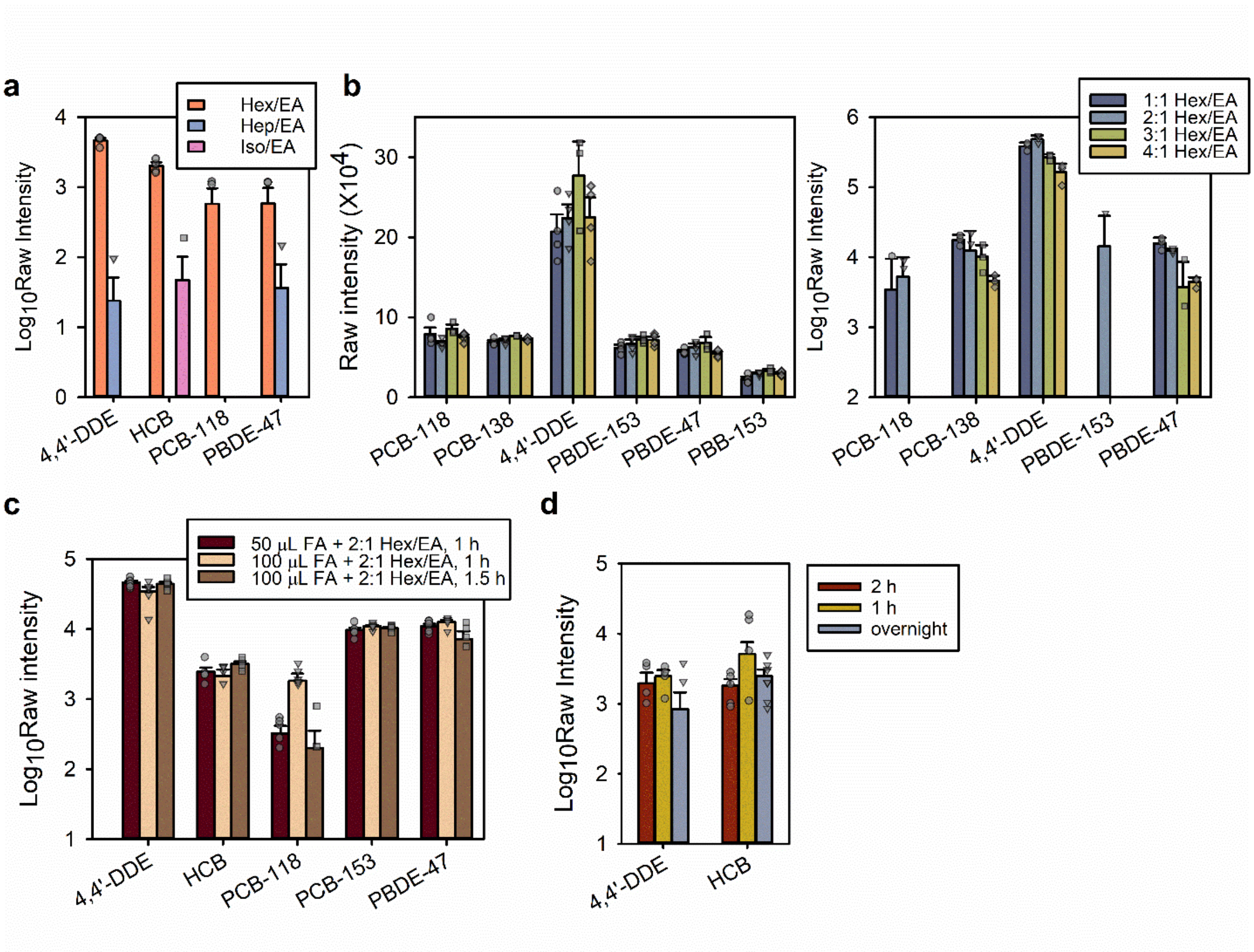

Supplemental Figure 1. Systematic variation of solvent composition, volume and extraction time was performed to optimize express liquid extraction (XLE) procedure. Raw intensities of top analytes were evaluated. Solvent composition was first determined by using 400 µL hexane (Hex), heptane (Hep) or isooctane (Iso) with 100 µL ethyl acetate (EA) in addition to 50 µL formic acid (FA) to analyze 200 µL Qstd3, a pooled EDTA plasma obtained from healthy donors (n= 50 biologically independent samples) purchased from Equitech-Bio (SHP45). Hex/EA extraction showed the highest intensities (n=4 independent experiments per condition) (**a**). Varying the ratio of Hex and EA in analysis of SRM-1958 (**b**, left, n=3 independent experiments for 3:1 Hex/EA and n=4 independent experiments for 1:1, 2:1 and 4:1 Hex/EA) and a non-fortified human plasma sample randomly selected from CHDWB archival biobank (**b**, right, n=3 independent experiments per condition) showed 2:1 ratio of Hex and EA was optimal. Next, we varied the volume of formic acid. Analysis of a non-fortified human plasma sample randomly selected from CHDWB archival biobank did not show significant differences (n=6 independent experiments per condition). The volume of Hex and EA was varied every 100 µL from 100 to 400 µL and determined based on the minimal volume of solvent (i.e., 200 µL) that gave a clear separation of organic phase to be transferred to a new vial (**c**). To avoid potential losses of acid-labile chemicals, we decided to use 50 µL formic acid. Ice-chilled extractions of 1 h versus 1.5 h in CHDWB plasma analysis (**c**) and 1 h, 2 h and overnight in analyzing a sample of human lung (**d**, obtained from lung transplant donation) were compared showing 1 h provided

optimal results and longer extraction time may cause degradation of chemicals (n=5 independent experiments for 2 h, n=4 independent experiments for 1 h and n=6 independent experiments for overnight). Data are presented as mean values ± SEM for all panels.

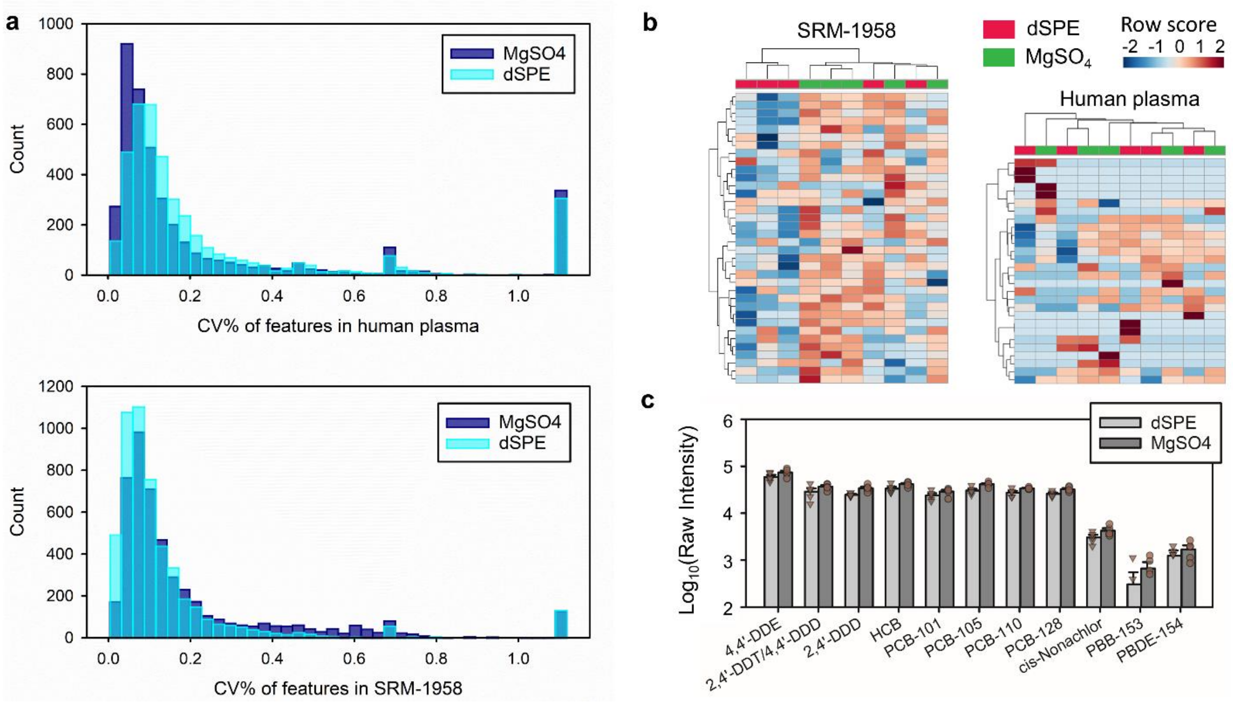

Supplementary Figure 2. Comparison of results from express liquid extraction (XLE) using high purity MgSO4 and QuEChERS dispersive solid phase extraction (dSPE). Results showed similar CV distribution in raw intensities of noise-filtered (>5 fold of isooctane blanks) features in pooled human plasma (n=5 independent experiments) and SRM-1958 (n=5 independent experiments) (**a**). Targeted analysis of chemicals showed more consistent peak intensities were found with $MgSO_4$ in SRM-1958 extraction by unsupervised hierarchical clustering (**b**). Most chemicals had similar average peak intensities with the two cleaning methods in SRM-1958 (<25% difference). The chemicals showing >25% difference all had higher intensities with $MgSO_4$. Data are presented as mean values ± SEM (n=5 independent experiments per condition) (**c**).

a

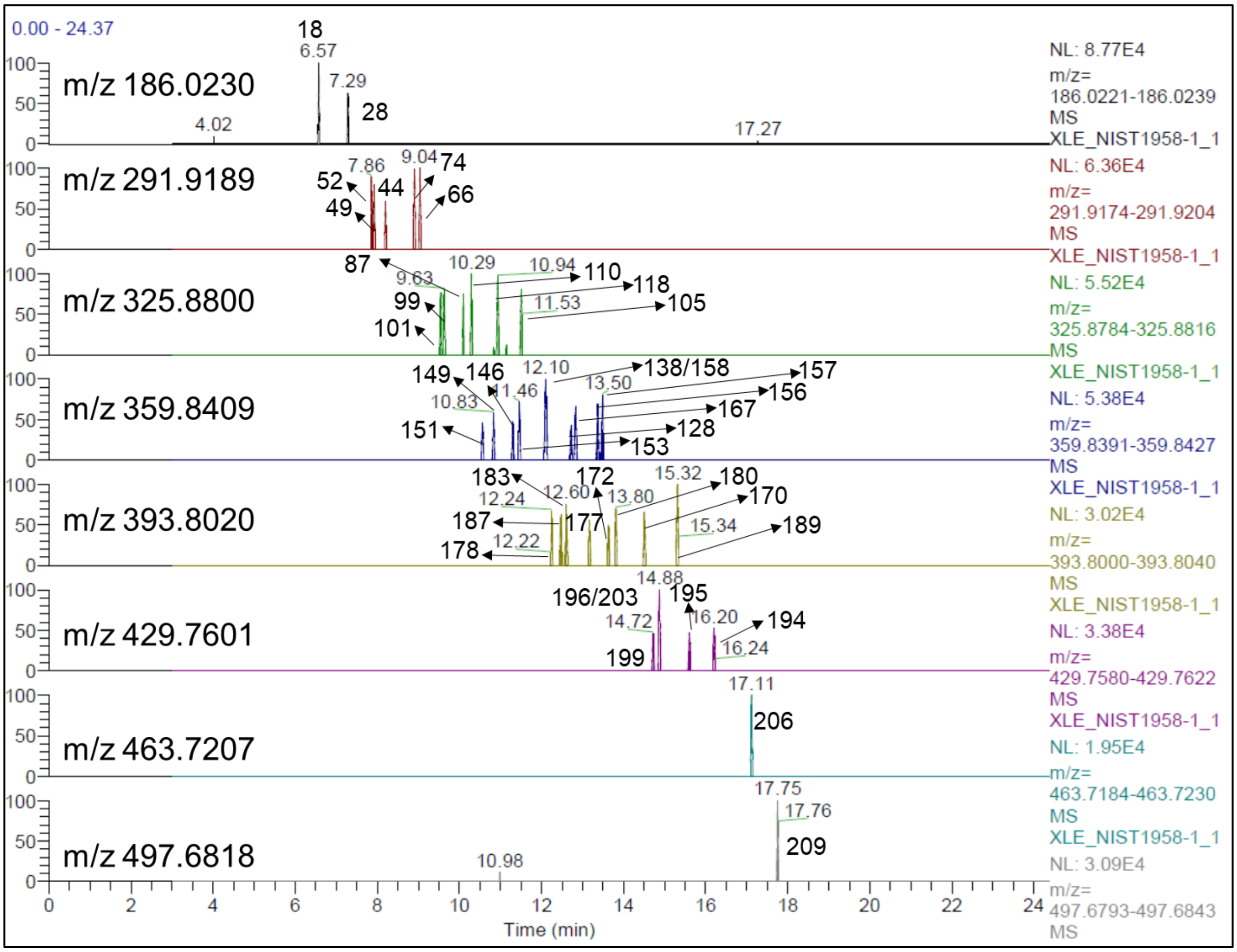

Supplementary Figure 3. Detected chromatographic traces of chemicals in NIST SRM-1958 using express liquid extraction (XLE) with gas chromatography high-resolution mass spectrometry (GC-HRMS). Chemicals include polychlorinated biphenyls (PCBs, **a**), chlorinated pesticides (**b**) and polybrominated diphenyl ethers (PBDEs) and polybrominated biphenyl (PBB) (**c**) in NIST1958 using XLE.

b

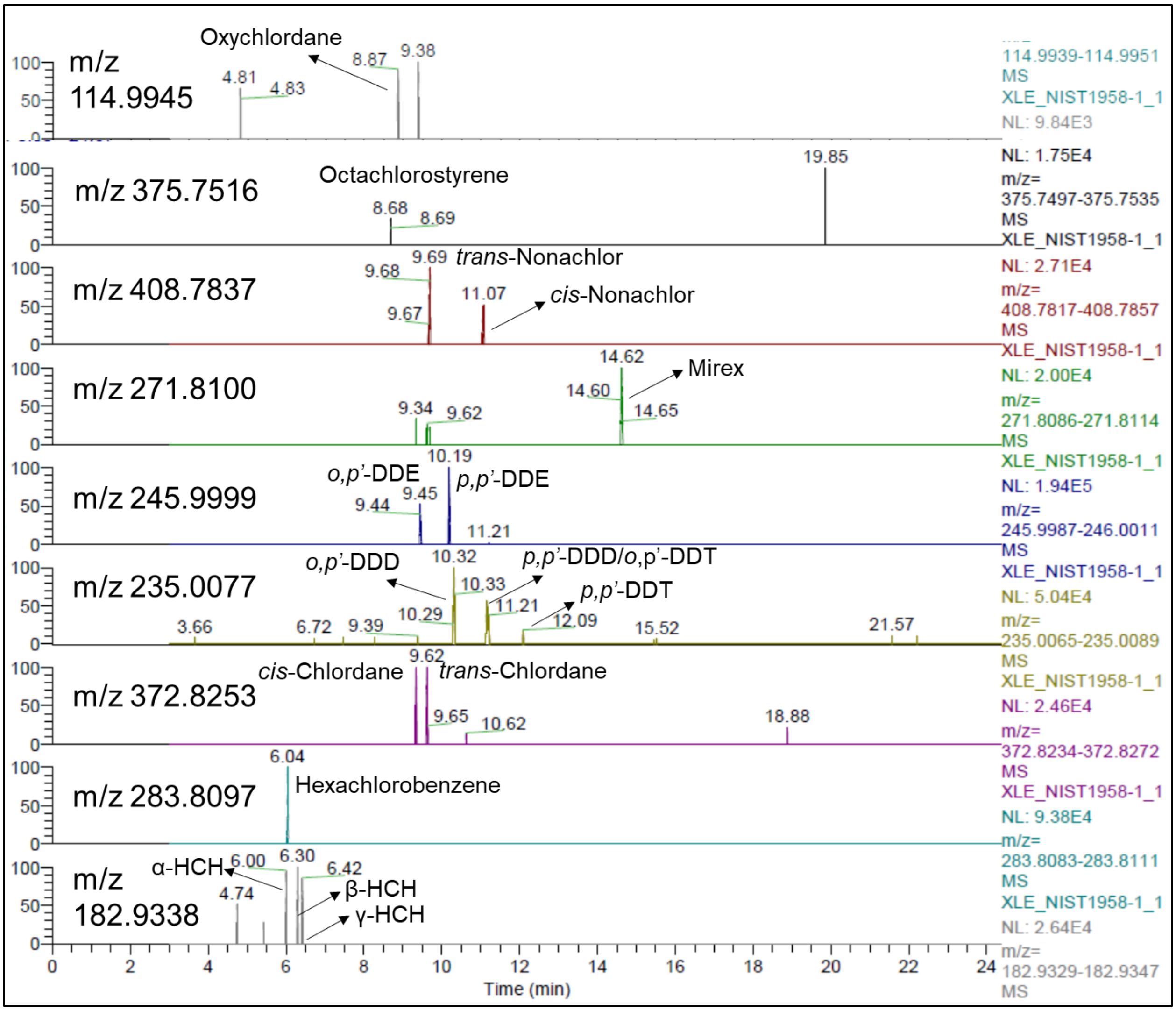

Continued: Supplementary Figure 3

c

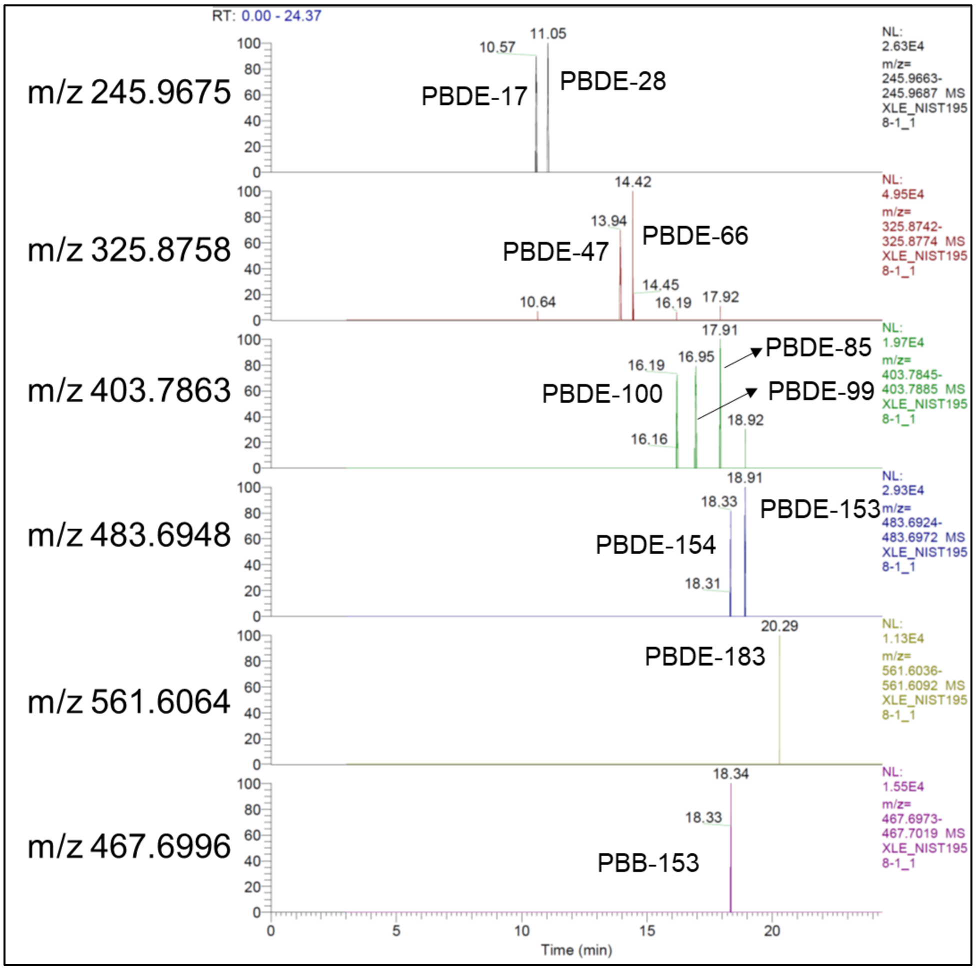

Continued: Supplementary Figure 3

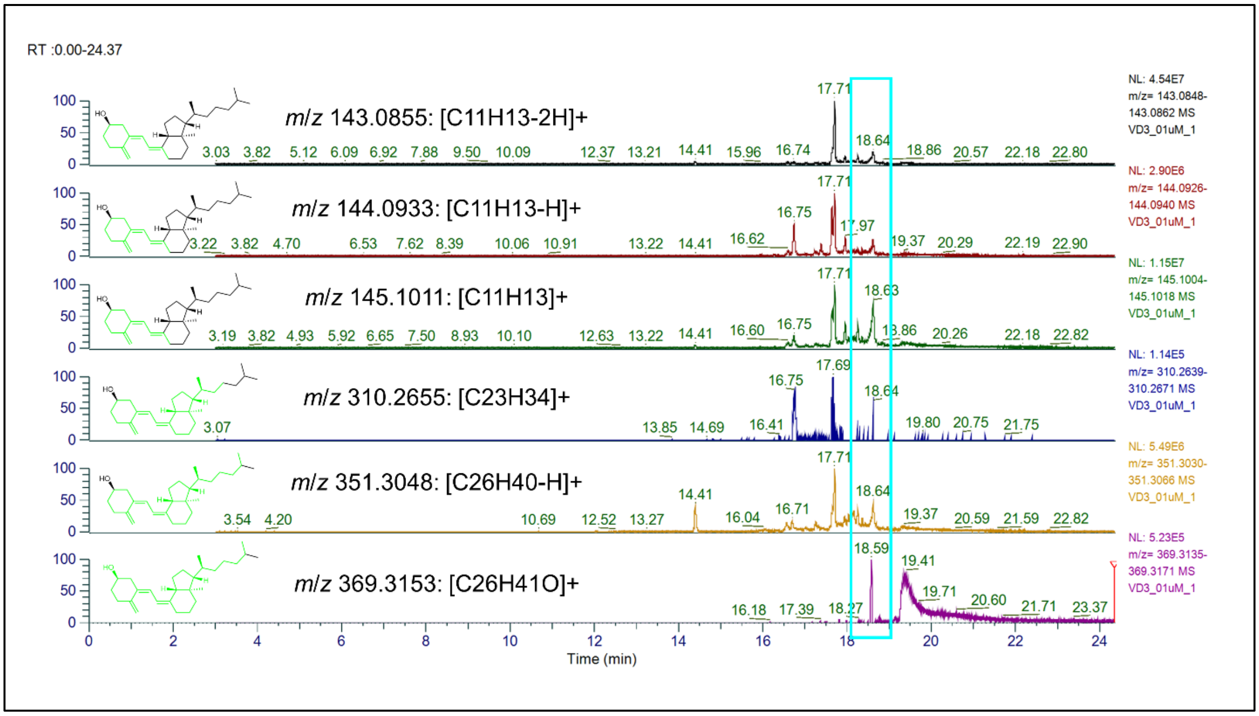

Supplementary Figure 4. Extracted ion chromatogram of *m/z* and retention time detected for cholecalciferol (0.1 μM in isooctane). Cyan box shows signal of various spectral *m/z* at consistent retention time of 18.6 min. Green colored structure represented predicted chemical fragments.

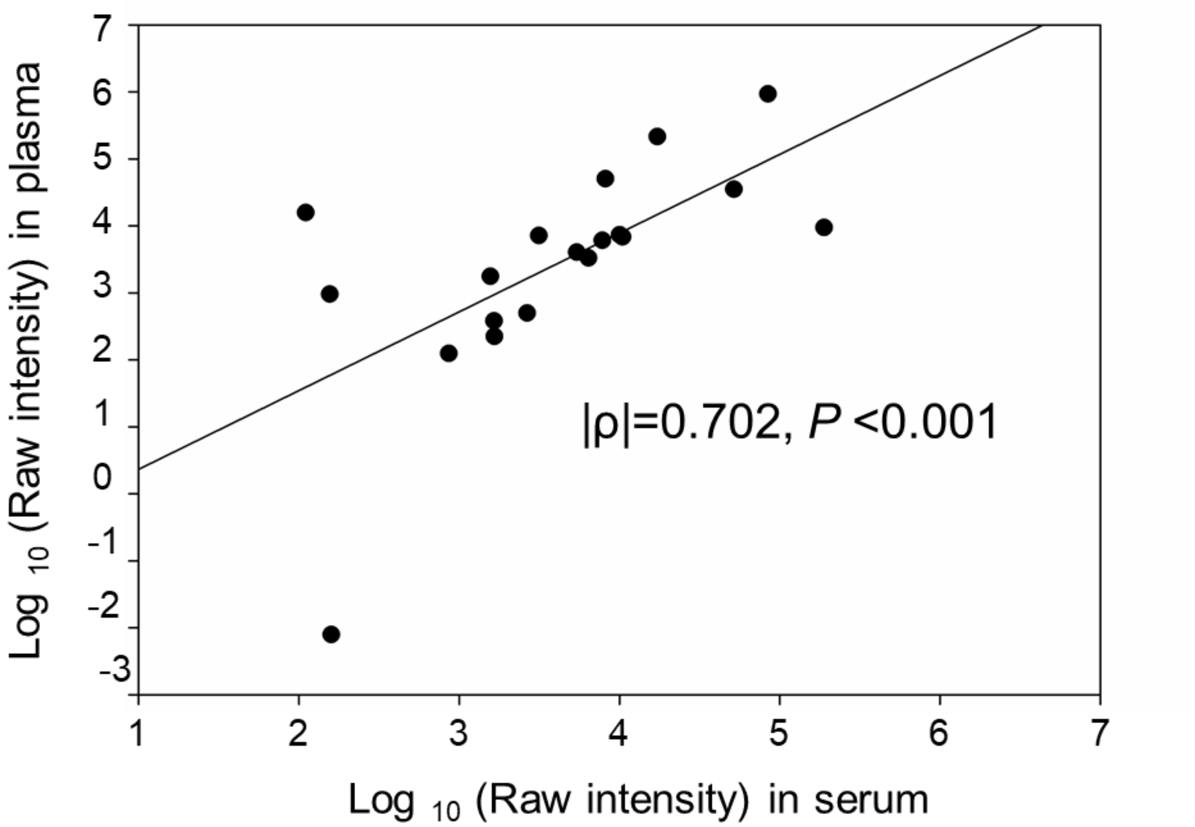

Supplementary Figure 5. Detected raw intensity of 4,4'-DDE (m/z = 245.9999, retention time 609 sec) randomly selected serum and EDTA-treated plasma collected from the same individuals (n=19 biologically independent samples). Spearman Rank Order Correlation shows a correlation coefficient of 0.702. Two-sided *t* test of the coefficient ($t = \rho\sqrt{\frac{n-2}{1-\rho^2}}$) showed *P* <0.001.
