# Supplementary material for: A scalable workflow to characterize the human exposome
Source: Nat Commun. 2021 Sep 22;12:5575. doi: 10.1038/s41467-021-25840-9 (PMC8458492; doi:10.1038/s41467-021-25840-9)
Supplement: Supplementary file 3 — Description of Additional Supplementary Files [file 41467_2021_25840_MOESM3_ESM.docx]

File Name: Supplementary Data 1
Description: Quantification of chemicals with reference values (certified and noncertified) in reconstituted standard reference material of human serum, NIST SRM-1958 and non-fortified reference material SRM-1957. Quantified levels and reference levels are presented in mass fraction values. Quantified values are presented in mean and standard error (SE) measure from n=9 repeated analysis of SRM-1958 and SRM-1957. Details of the reference values can be found in SRM-1958 and SRM-1957 certificates. N.D. = Not detectable, defined by no intensity extracted from the raw data.

File Name: Supplementary Data 2

Description: Comparison of quantification results in n=20 human plasma samples, by reference standardization using SRM-1958 and by external standard curves (0-2 ng/mL). Results of standard curve were not corrected by recovery of [^13^C] labelled internal standards. Values of quantification are presented in ng/kg. Spearman's correlation coefficient of each sample quantification was calculated.

File Name: Supplementary Data 3

Description: Comparison of quantification results in n=11 human lung samples, by reference standardization using SRM-1958 and by Response Factor (RF) calculated with spiked 13C-isoptoic standards. Quantification values are presented in ng/kg. Spearman's correlation coefficient of each sample quantification was calculated.

File Name: Supplementary Data 4

Description: Information of 378 chemical authentic standards on retention time, spectral information (the most abundant m/z: mz1 to mz5), linearity and limit of detection (LOD). The calibration curves were established over a range of 0.1 ng/mL to 20 ng/mL with a subset of chemicals re-run over 0.05 ng/mL to 1 ng/mL (indicated by *).

File Name: Supplementary Data 5

Description: Raw intensity and quantification of 80 human plasma samples collected via Center of Health Discovery and Well Being (CHDWB). Chemicals are sorted by the order from top to bottom in the heatmap of Figure 4a. Quantified levels were calculated by reference standardization using NIST SRM1958 and SRM 1957 (ng/g whole weight of plasma). For those chemicals that do not have reference values, quantification was not performed (marked N/A).

File Name: Supplementary Data 6

Description: Information of 305 non-halogenated chemical authentic standards on retention time, spectral information (the most abundant m/z: mz1 to mz4).

File Name: Supplementary Data 7

Description: Raw intensity of non-halogenated chemicals in NIST SRM1957, SRM1958 and 60 human plasma samples collected via Center of Health Discovery and Well Being (CHDWB). Chemicals are identified by 1) match to authentic standard library (Δm/z=5 ppm, ΔRT = 30 sec), 2) spectral clustering using RAMClustR that was based on similarity of retention time and intensity across samples among potential spectral m/z, and 3) primary ion (presented m/z in this table) were 10-fold higher than solvent blanks.

File Name: Supplementary Data 8

Description: Concentration (ng/g tissue weight) of chemicals measured by reference standardization in 11 human lungs. Chemicals in Fig 4b are sorted in the order of correlation clustering. Other chemicals were detected only in 1 out of 11 samples and were not included in Fig 4b.

File Name: Supplementary Data 9

Description: Concentration (ng/g tissue weight) of chemicals measured by reference standardization in 5 human thyroids. Chemicals in Fig 4b are sorted in the order of correlation clustering. Other chemicasl were detected only in 1 out of 5 samples and were not included in Fig 4b.

File Name: Supplementary Data 10

Description: Concentration (ng/kg wet weight) of chemicals measured by reference standardization in 6 human stool samples. Chemicals in Fig 4b are sorted in the order of correlation clustering. Other chemicals were detected only in 1 out of 6 samples and were not included in Fig 4b.

File Name: Supplementary Data 11

Description: Information of software used in data analysis including installation guide, code with parameter settings and example of output files.
