# Supplementary material for: A scalable workflow to characterize the human exposome
Source: Nat Commun. 2021 Sep 22;12:5575. doi: 10.1038/s41467-021-25840-9 (PMC8458492; doi:10.1038/s41467-021-25840-9)
Supplement: Supplementary file 14 — Dataset 11 [file 41467_2021_25840_MOESM14_ESM.zip › Code.docx]

**XCMS installation:** XCMS^1^ package (version 3.8.2) was downloaded from <https://github.com/sneumann/xcms>.

To install this package, enter the following in R:

if (!requireNamespace("BiocManager", quietly = TRUE))

install.packages("BiocManager")

BiocManager::install("xcms")

**Code for XCMS extraction**: The parameters of XCMS have been optimized based on 1) data extraction of targeted chemicals in authentic standard mixtures (200-300 chemicals per mixture) and those identified in SRM1957 and SRM1958; and 2) results from IPO package, a published tool for automated optimization of XCMS parameters ^2^. The XCMS parameters that generate fewer features (~20,000 before filtering as opposed to ~40,000) caused missing of narrow peaks (e.g. PBDE-153) and thus were not used. In addition, we found inclusion of an external standard that contain a range of targeted chemicals standards facilitates peak extraction of the smaller and narrow peaks. Occasionally, the extremely narrow peaks may be missed by the extraction and will need manual integration or extraction with narrower peak width parameters.

library(xcms)

library(doParallel)

library(BiocParallel)

ptm <- proc.time()

#use n-2 for number of cores

c1<-4

num_files<- length(ms_files)

print(paste("Number of cores: ", c1, sep= ""))

print(paste("Number of files to be proccessed: ", num_files, sep= ""))

#create output directory

dir.create(out_dir, showWarnings= TRUE)

setwd(out_dir)

#create initial xcms set

step_01_set<- xcmsSet(files= ms_files, method= "centWave", BPPARAM = MulticoreParam(workers = c1), ppm= 2.5, peakwidth=c(2,30), snthr=1, mzdiff=-0.001, noise=1000, prefilter= c(2,100))

save.image(paste(out_dir, "step01_xcms_results_image.RData", sep="/"))

saveRDS(step_01_set, 'step01_set.rds')

step_02_set<- group(step_01_set, method="density", bw=2, mzwid=0.01, max=100, minsamp = 1, minfrac=0.01)

save.image(paste(out_dir, "step02_xcms_results_image.RData", sep="/"))

saveRDS(step_02_set, 'step02_set.rds')

#retention time correction

pdf("Deviation_plot.pdf")

#step_03_set<- retcor(step_02_set, missing= round(0.5*num_files,0), smooth="linear", plottype="deviation")

step_03_set<- retcor(step_02_set, missing= round(0.6*num_files,0), smooth="linear", plottype="deviation")

save.image(paste(out_dir, "step03_xcms_results_image.RData", sep="/"))

saveRDS(step_03_set, 'step03_set.rds')

#peak grouping post retention time correction

step_04_set<- group(step_03_set, method="density", bw=2, mzwid=0.015, max=100, minsamp = 1, minfrac=0.01)

save.image(paste(out_dir, "step04_xcms_results_image.RData", sep="/"))

saveRDS(step_04_set, 'step04_set.rds')

#fill missing values and create final object

xset_final<- fillPeaks(object=step_04_set, method= "chrom", BPPARAM = MulticoreParam(workers = c1))

save.image(paste(out_dir, "final_xcms_results_image.RData", sep="/"))

saveRDS(xset_final, 'final_set.rds')

#create feature table from xcms set

feature_table<- peakTable(object=xset_final)

num_peaks<- nrow(feature_table)

#write featute table to out_dir

write.table(feature_table, paste(out_dir, "xcms_feature_table.txt", sep="/"), sep= "\t", row.names=FALSE)

print("xcms processing complete")

print(paste("Number of peaks detected: ", num_peaks, sep= ""))

proc.time() - ptm

**xMSanalyzer installation:** xMSanalyzer ^3^ (version 2.0.6.1) was downloaded from <https://rdrr.io/github/kuppal2/xMSanalyzer/>.

Install the latest version of this package by entering the following in R:

install.packages("remotes")

remotes::install_github("kuppal2/xMSanalyzer")

**Code for using xMSanalyzer for targeted search in chemical database**: We use GetVenn function in xMSanalyzer R package^3^ to select features that matched the m/z spectra and retention time of targeted chemicals. A tolerance of Δm/z=5 ppm and ΔRT = 30 seconds was used for this study.

library(xMSanalyzer)

##### the path of table1 ######

table1="~/XinHu/GC_xcms/Clustered_nonhalo_batch3to5.txt"

##### the path of table2 ######

table2="~/XinHu/GC_xcms/target_list_nonhalogenated_04 29 2021.txt"

##### the path of output ######

outloc="~/XinHu/GC_xcms/"

######### mz tolerance ########

mz_diff=5 #5ppm

######### time tolerance ########

time_diff=30

###############################

table1=read.table(table1,sep="\t",header=TRUE)

table2=read.table(table2,sep="\t",header=TRUE)

colnames(table1)[1:2]<-c("table1_mz","table1_time")

colnames(table2)[1:2]<-c("table2_mz","table2_time")

overlap<-getVenn(table1,"A",table2,"B",mz.thresh =mz_diff, time.thresh=time_diff,alignment.tool=NA,xMSanalyzer.outloc="NA",use.unique.mz=FALSE,plotvenn=FALSE)

unlink("NA", recursive = TRUE, force = FALSE)

if(dim(overlap$common)[1]>0){

combine=cbind(table1[overlap$common$index.A,],table2[overlap$common$index.B,])

write.table(combine,paste(outloc,"merged_table.csv",sep=""),sep=",",row.names=FALSE)

}

**RAMClustR installation:** RAMClustR ^4^ package (version 1.1.0) was downloaded from <https://github.com/cbroeckl/RAMClustR>.

To install from R console:

install.packages("devtools", repos="http://cran.us.r-project.org", dependencies=TRUE)

library(devtools)

install_github("cbroeckl/RAMClustR", build_vignettes = TRUE, dependencies = TRUE)

**Code for RAMClustR for targeted analysis**: The parameters of RAMclustR have been optimized based on clustering accuracy of chemicals reported in SRM-1958. We found that larger sample size (at least 3 batches of 20 samples, triple injections) enhances the accuracy of spectra clustering.

library(RAMClustR)

experiment <- defineExperiment(force.skip = TRUE)

RC1 <- ramclustR(ms = paste0(getwd(), "/batch8_Ramclustr.csv"), featdelim = "_", st=1, sr=0.5, ExpDes=experiment, sampNameCol = 1,minModuleSize=3, normalize = "none")

write.csv(RC1$featclus, file="featclus_CHDWB_8.csv", row.names=TRUE)

write.csv(RC1$MSdata, file="msdata_order_CHDWB_8.csv", row.names=TRUE)

**Code for RAMClustR for untargeted analysis**

library(RAMClustR)

experiment <- defineExperiment(csv = TRUE)

#experiment <- defineExperiment(force.skip = TRUE)

RC1 <- ramclustR(ms = paste0(getwd(), "/humanlung_RT1080to1200.csv"), featdelim = "_", st=1, sr=0.5, ExpDes=experiment, sampNameCol = 1,minModuleSize=3, normalize = "none")

write.csv(RC1$featclus, file="featclus_untargeted_900to1200.csv", row.names=TRUE)

write.csv(RC1$MSdata, file="msdata_order_untargeted_900to1200.csv", row.names=TRUE)

write.csv(RC1$SpecAbund, file="SpecAbund_order_untargeted_900to1200.csv", row.names=TRUE)

The generated .msp files from RAMClustR can be imported into MS-Finder or XCalibur for library search.

**Code for generation figures:** For R generated figures, we used “Hmisc” version 4.0.3 (<https://cran.r-project.org/web/packages/Hmisc/index.html>), “ggplot2” version 2.2.1 (<https://ggplot2.tidyverse.org/>), “wesanderson” version 0.3.6 (<https://cran.r-project.org/web/packages/wesanderson/index.html>). Input data is provided in Source Data.

To install, enter the following in R:

install.packages("Hmisc")

install.packages("wesanderson")

install.packages("ggplot2")

**Code for correlation heatmap**

library("Hmisc")

my_data1 <- read.table ( paste(dir, sep = "","/quanti_plasma.txt"), check.names = F,fill = TRUE,header = TRUE,sep = "\t")

corr <- round(cor(as.matrix(my_data1[,2:46])), 2)

corr2 <- round(cor(as.matrix(my_data1[,2:46]),method="spearman"), 2)

res1<-rcorr(as.matrix(my_data1[2:81,2:46]))

# ++++++++++++++++++++++++++++

# flattenCorrMatrix

# ++++++++++++++++++++++++++++

# cormat : matrix of the correlation coefficients

# pmat : matrix of the correlation p-values

flattenCorrMatrix <- function(cormat, pmat) {

ut <- upper.tri(cormat)

data.frame(

row = rownames(cormat)[row(cormat)[ut]],

column = rownames(cormat)[col(cormat)[ut]],

cor =(cormat)[ut],

p = pmat[ut]

)

}

corr_data<-flattenCorrMatrix(res1$r, res1$P)

# Heatmap

library(ggplot2)

ggplot(data = corr_data, aes(column, row, fill = cor))+

geom_tile(color = "white")+

scale_fill_gradient2(low = "blue", high = "red", mid = "white",

midpoint = 0, limit = c(-1,1), space = "Lab",

name="Pearson\nCorrelation") +

theme_minimal()+

theme(axis.text.x = element_text(angle = 45, vjust = 1,

size = 12, hjust = 1))+

coord_fixed()

#helper function

reorder_cormat <- function(cormat){

# Use correlation between variables as distance

dd <- as.dist((1-cormat)/2)

hc <- hclust(dd)

cormat <-cormat[hc$order, hc$order]

}

# Reorder the correlation matrix

cormat2 <- reorder_cormat(corr2)

# Melt the correlation matrix

melted_cormat2 <- melt(cormat2, na.rm = TRUE)

# Create a ggheatmap

ggheatmap2 <- ggplot(melted_cormat2, aes(Var2, Var1, fill = value))+

geom_tile(color = "white", lwd=0.5)+

scale_fill_gradient2(low = "#003399", high = "#660000", mid = "white",

midpoint = 0, limit = c(-1,1),space = "Lab",

name="Pearson\nCorrelation") +

theme(axis.text=element_blank(),axis.ticks=element_blank(),axis.title=element_blank())+ coord_fixed()

print(ggheatmap2)

ggheatmap3 <- ggplot(melted_cormat2, aes(Var2, Var1, fill = value))+

geom_tile(color = "white", lwd=0.5)+

scale_fill_gradient2(low = "#003399", high = "#660000", mid = "white",

midpoint = 0, limit = c(-1,1),space = "Lab",

name="Pearson\nCorrelation") +

theme_minimal()+ coord_fixed()

print(ggheatmap3)

**Code for violin plot**

library(ggplot2)

p <- ggplot(my_data, aes(x=chemical, y=log10(conc)))+geom_violin(trim = FALSE, lwd=0, scale="width",fill="deepskyblue")+theme(legend.position = "none")+theme(axis.line=element_line(colour="black",size=0.8),panel.background=element_rect(fill="white",colour=NA),

axis.text=element_text(family="Arial", colour="black",size=18),axis.title=element_blank()) + geom_point(pch = 16, size =0.6, position = "jitter") +stat_summary(fun.y = mean, fun.ymin = mean, fun.ymax = mean,

geom = "crossbar",

width = 0.2,lwd=0.4,colour="white"

)

**Code for bubble plot**

library(ggplot2)

my_data2 <- read.table ( paste(dir, sep = "","/untargeted_specabun_lung_1080to1200.txt"), check.names = F,fill = TRUE,header = TRUE,sep = "\t")

library("wesanderson")

pal <- wes_palette("Zissou1", 573, type = "continuous")

#show cluster position

p_clu <- ggplot(my_data2,aes(x=time,y=mz))+geom_point(aes(colour = cluster,size=log10(Intensity)))+

scale_colour_gradientn(colours = pal)+scale_size_continuous(range=c(0.2,7))+

geom_point(aes(size=log10(Intensity),alpha = factor(edge)),shape = 1, stroke=1)+

theme(axis.line=element_line(colour="black"),panel.background=element_rect(fill="white",colour=NA),

axis.text=element_text(family="Arial", colour="black",size=14),axis.title=element_blank())

p_clu

#final figure

p <- ggplot(my_data2,aes(x=time,y=mz))+geom_point(aes(fill = cluster,size=log10(Intensity)),shape=21)+

scale_fill_gradientn(colours = pal)+ scale_size_continuous(range=c(0.1,4))+

theme(axis.line=element_line(colour="black"),panel.background=element_rect(fill="white",colour=NA),

axis.text=element_text(family="Arial", colour="black",size=14),axis.title=element_blank())

**Reference**

1. Smith CA, Want EJ, O'Maille G, Abagyan R, Siuzdak G. XCMS: processing mass spectrometry data for metabolite profiling using nonlinear peak alignment, matching, and identification. *Anal Chem* **78**, 779-787 (2006).

2. Libiseller G*, et al.* IPO: a tool for automated optimization of XCMS parameters. *BMC Bioinformatics* **16**, 118 (2015).

3. Uppal K*, et al.* xMSanalyzer: automated pipeline for improved feature detection and downstream analysis of large-scale, non-targeted metabolomics data. *BMC Bioinformatics* **14**, 15 (2013).

4. Broeckling CD, Afsar FA, Neumann S, Ben-Hur A, Prenni JE. RAMClust: a novel feature clustering method enables spectral-matching-based annotation for metabolomics data. *Anal Chem* **86**, 6812-6817 (2014).
