# Supplementary material for: A scalable workflow to characterize the human exposome
Source: Nat Commun. 2021 Sep 22;12:5575. doi: 10.1038/s41467-021-25840-9 (PMC8458492; doi:10.1038/s41467-021-25840-9)
Supplement: Supplementary file 15 — Reporting Summary [file 41467_2021_25840_MOESM15_ESM.pdf]

nature research

Corresponding author(s): Dean P. Jones

Last updated by author(s): 8/6/2021

Reporting Summary

Nature Research wishes to improve the reproducibility of the work that we publish. This form provides structure for consistency and transparency in reporting. For further information on Nature Research policies, see our Editorial Policies and the Editorial Policy Checklist.

Statistics

For all statistical analyses, confirm that the following items are present in the figure legend, table legend, main text, or Methods section.

n/a	Confirmed	
☐	☒	The exact sample size (*n*) for each experimental group/condition, given as a discrete number and unit of measurement
☐	☒	A statement on whether measurements were taken from distinct samples or whether the same sample was measured repeatedly
☐	☒	The statistical test(s) used AND whether they are one- or two-sided<br>*Only common tests should be described solely by name; describe more complex techniques in the Methods section.*
☒	☐	A description of all covariates tested
☐	☒	A description of any assumptions or corrections, such as tests of normality and adjustment for multiple comparisons
☐	☒	A full description of the statistical parameters including central tendency (e.g. means) or other basic estimates (e.g. regression coefficient) AND variation (e.g. standard deviation) or associated estimates of uncertainty (e.g. confidence intervals)
☐	☒	For null hypothesis testing, the test statistic (e.g. *F*, *t*, *r*) with confidence intervals, effect sizes, degrees of freedom and *P* value noted<br>*Give P values as exact values whenever suitable.*
☒	☐	For Bayesian analysis, information on the choice of priors and Markov chain Monte Carlo settings
☒	☐	For hierarchical and complex designs, identification of the appropriate level for tests and full reporting of outcomes
☐	☒	Estimates of effect sizes (e.g. Cohen's *d*, Pearson's *r*), indicating how they were calculated

Our web collection on statistics for biologists contains articles on many of the points above.

Software and code

Policy information about availability of computer code

Data collection	Mass spectral data was acquired using TraceFinder v4.1 (ThermoFisher Scientific).
Data analysis	Data was extracted using XCMS (R package version 3.8.2). Targeted analysis was performed by search against in-house chemical library using GetVenn function in xMSAnalyzer (R package). Spectral clusters were generated by RAMClustR (R package). Untargeted analysis was performed by spectral clustering using RAMClustR followed by library search using MS-Finder (v3.42) and XCalibur (v 4.2.1). We did not develop custom codes for this work. Rather, we used existing softwares. The code provided with the manuscript is simply script of using the software to show the parameter settings, and was provided in the form of supplementary information.

For manuscripts utilizing custom algorithms or software that are central to the research but not yet described in published literature, software must be made available to editors and reviewers. We strongly encourage code deposition in a community repository (e.g. GitHub). See the Nature Research guidelines for submitting code & software for further information.

Data

Policy information about availability of data

All manuscripts must include a data availability statement. This statement should provide the following information, where applicable:

- Accession codes, unique identifiers, or web links for publicly available datasets
- A list of figures that have associated raw data
- A description of any restrictions on data availability

The raw data generated in this study have been deposited in the National Institutes of Health (NIH) Common Fund's National Metabolomics Data Repository (NMDR), the Metabolomics Workbench (https://www.metabolomicsworkbench.org) under assigned Project ID PR001136. The data can be fully accessed via its Project DOI: http://dx.doi.org/10.21228/M8VQ4D. The Metabolomics Workbench is supported by NIH grant U2C-DK119886. The processed data are provided in the Supplementary Information/Source Data file.

Field-specific reporting

Please select the one below that is the best fit for your research. If you are not sure, read the appropriate sections before making your selection.

[x] Life sciences   [ ] Behavioural & social sciences   [ ] Ecological, evolutionary & environmental sciences

For a reference copy of the document with all sections, see nature.com/documents/nr-reporting-summary-flat.pdf

Life sciences study design

All studies must disclose on these points even when the disclosure is negative.

Sample size	At initial stage of XLE development using aliquots of standard reference material of human serum and pooled human plasma, we have tested and decided n=4 is sufficient to compare XLE to LLE and SPE methods. More experimental replicates were added to get n=9-16 for more accurate estimate of quantitative measurements. As a pilot to demonstrate utility of XLE in human biospecimens, n=80 were used to analyze archival plasma samples; n=11 and 5 were used to analyze donated lungs and thyroids and the sample size was determined by availability of the organs.
Data exclusions	No data was excluded.
Replication	XLE procedure experiments were repeated 2-3 times at sets of n=3-5 and representative results were presented. Recovery of standards and quantification of chemicals in SRM were repeated two times and the results were included and combined for a total of n=9-16, all of which were presented. Reference standardization validation in human plasma was replicated with a different subset of samples. Quantification of chemicals in a subset of 2 lung samples were repeated twice. All replications generated similar results.
Randomization	Samples of human biospecimens were randomized and allocated to batches for instrumental analysis.
Blinding	The investigators were blinded to the demographic information of human participants.

Reporting for specific materials, systems and methods

We require information from authors about some types of materials, experimental systems and methods used in many studies. Here, indicate whether each material, system or method listed is relevant to your study. If you are not sure if a list item applies to your research, read the appropriate section before selecting a response.

Materials & experimental systems

n/a	Involved in the study
[x]	[ ] Antibodies
[x]	[ ] Eukaryotic cell lines
[x]	[ ] Palaeontology and archaeology
[x]	[ ] Animals and other organisms
[ ]	[x] Human research participants
[x]	[ ] Clinical data
[x]	[ ] Dual use research of concern

Methods

n/a	Involved in the study
[x]	[ ] ChIP-seq
[x]	[ ] Flow cytometry
[x]	[ ] MRI-based neuroimaging

Human research participants

Policy information about studies involving human research participants

Population characteristics	Plasma samples from 80 individuals without known disease were randomly selected from archival samples obtained from the Center for Health Discovery and Well Being (CHDWB) cohort of approximately 750 individuals including both genders and caucasian, african american, hispanic and asian. Among the randomly selected 80 individuals, 57 were females and 23 were males ageing between 41 to 68. Primary outcomes of this cohort has been published.
Recruitment	The inclusion and exclusion criteria of CHDWB were structured to identify a cohort of adults with few known acute or uncontrollable chronic conditions. The CHDWB cohort represents a healthy population relative to the characteristics of the general US population. Individuals were excluded if they were functionally impaired by poorly controlled chronic disease or acute illness, but included if taking medications for common ailments. Plasma samples were collected at the time of recruitment (baseline), six and 12 months later. Health partners of the Center provide personalized counselling regarding promotion of a healthier lifestyle; yet all health behavior changes (if any) are entirely at the discretion of the individual.<br>For posttransplant human donor lungs, de-identified tissue was collected by the Emory Transplant Center under Institutional Review Board approved tissue-acquisition protocols (IRB approval No. 00006248) and Cystic Fibrosis Biospecimen Registry at Emory University (IRB approval No. 00095116). Potential biases are that the levels of chemicals in the CHDWB cohort only reflect exposure levels in relatively healthy individuals of 40 and older, while the levels in postmortem lungs and thyroids reflect exposure levels in elderly due to the organ sources.
Ethics oversight	The recruitment strategy, consent forms, and data collection protocols were approved by the Emory University institutional review board (IRB approval No. 00007243). The informed consent contains wording to allow investigators to perform measurements and access to the participant's health-related data for research purposes at the discretion of the investigators or Center staff and thus the present study. For posttransplant human donor lungs, de-identified tissue was collected by the Emory Transplant Center under Institutional Review Board approved tissue-acquisition protocols (IRB approval No. 00006248) and Cystic Fibrosis Biospecimen Registry at Emory University (IRB approval No. 00095116) with patient informed consent, which gives permission to use discarded tissues from organ transplant patients for assay development and thus the present study. Collection and research using postmortem organs (i.e. the 6 lungs and all thyroids in this study) do not represent human research activity since the samples are not from living individuals, and thus waiver of patient consent has been approved by Emory Institutional Review Board. The use of stool samples from patients with primary sclerosing cholangitis (PSC) in the current study was approved (IRB 14-008752; Mayo Clinic, Rochester, MN) and additional informed consent was waived in accordance with 45 CFR 46.116, as the study is of minimal risk to subjects.

Note that full information on the approval of the study protocol must also be provided in the manuscript.
